# Synergistic Effects of FGF-18 and TGF-*β*3 on the Chondrogenesis of Human Adipose-Derived Mesenchymal Stem Cells in the Pellet Culture

**DOI:** 10.1155/2018/7139485

**Published:** 2018-05-09

**Authors:** Liangjie Huang, Lingxian Yi, Chunli Zhang, Ying He, Liangliang Zhou, Yan Liu, Long Qian, Shuxun Hou, Tujun Weng

**Affiliations:** ^1^Department of Orthopaedics, First Affiliated Hospital of PLA General Hospital, No. 51 Fucheng Road, Beijing, China; ^2^Department of Orthopaedics, The 41st Hospital of PLA, No. 80 Naidong Road, Shannan, China; ^3^Department of ICU, The 306th Hospital of PLA, No. 9 Anxiangbeili Road, Beijing, China

## Abstract

Cell-based therapy serves as an effective way for cartilage repair. Compared with a limited source of autologous chondrocytes, adipose-derived stem cells (ADSCs) are proposed as an attractive cell source for cartilage regeneration. How to drive chondrogenic differentiation of ADSCs efficiently remains to be further investigated. TGF-*β*3 has shown a strong chondrogenic action on ADSCs. Recently, fibroblast growth factor 18 (FGF-18) has gained marked attention due to its anabolic effects on cartilage metabolism, but existing data regarding the role of FGF-18 on the chondrogenic potential of mesenchymal stem cells (MSCs) are conflicting. In addition, whether the combined application of FGF-18 and TGF-*β*3 would improve the efficiency of the chondrogenic potential of ADSCs has not been thoroughly studied. In the current study, we isolated human ADSCs and characterized the expression of their surface antigens. Also, we evaluated the chondrogenic potential of FGF-18 on ADSCs using an *in vitro* pellet model by measuring glycosaminoglycan (GAG) content, collagen level, histologic appearance, and expression of cartilage-related genes. We found that FGF-18, similarly to TGF-*β*3, had a positive impact on chondrogenic differentiation and matrix deposition when presented throughout the culture period. More importantly, we observed synergistic effects of FGF-18 and TGF-*β*3 on the chondrogenic differentiation of ADSCs in the *in vitro* pellet model. Our results provide critical information on the therapeutic use of ADSCs with the help of FGF-18 and TGF-*β*3 for cartilage regeneration.

## 1. Introduction

Articular cartilage is an avascular tissue with a poor capacity for regeneration and repair [1]. Cartilage defects often induce the development of osteoarthritis and consequent disability. Autologous chondrocyte implantation has proven beneficial for treating articular defects in patients during the last two decades [2, 3]. However, difficulties in the expansion of chondrocytes and proneness to dedifferentiate during extended cultures have led to the use of mesenchymal stem cells (MSCs) as an alternative for cartilage repair [4].

Adult MSCs display high potential for proliferation and multipotency and can be isolated from various tissues such as bone marrow and adipose tissue [4]. The capacity of self-renewal and the chondrogenic potential of human bone marrow-derived MSCs (BMSCs) have been well documented [5]. Compared with BMSCs, human adipose-derived stem cells (ADSCs) contain similar characteristics as BMSCs and have their own advantages in abundance, ease of access, and better maintenance of cell phenotype during passage [6]. Growing evidence has suggested that bone marrow and adipose-derived stem cells could promote the regeneration of cartilage; however, underlying mechanisms that govern their chondrogenic potential remain largely unknown [4]. Therefore, it is crucial to identify key growth factors that will selectively promote chondrogenesis and cartilage production in the microenvironment.

Since mesenchymal condensation is critical in cartilage development, high-density cell pellets that mimic the chondrogenic program were developed and used to examine the chondrogenic differentiation of MSCs [7]. Specific growth factors or bioactive molecules were added into the pellet culture system to determine the effects in the chondrogenic differentiation for MSCs. Presently, members of the transforming growth factor-*β* (TGF-*β*) superfamily are well recognized as the main chondroinductive growth factors [8]. There are three members of TGF-*β*s, including TGF-*β*1, TGF-*β*2, and TGF-*β*3, which bind with the TGF-*β* receptor then activate downstream molecules. Among these, TGF-*β*3 has the strongest chondrogenic effects on ADSCs, showing an increased matrix deposition and chondrogenic differentiation [9, 10].

Recently, fibroblast growth factors (FGFs) and their receptors have been found to play a critical role in articular cartilage repair and the maintenance of cartilage [11]. Among these members, FGF-18 has acquired marked attention because of its anabolic effects on cartilage [12]. It has been found that FGF-18 can promote the development of cartilage, delay the degeneration of articular cartilage, and stimulate the potency for the regeneration of hyaline articular cartilage [13–16]. However, existing data regarding the effects of FGF-18 on the chondrogenic potential of MSCs are conflicting. In limb bud mesenchymal cells, it has been suggested that FGF-18 through selectively binding with FGFR3 promotes the differentiation of prechondrogenic mesenchymal cells to cartilage-producing chondrocytes [13]. On the other hand, FGF-18 exhibited a negative role on the chondrogenesis of human BMSCs when added at the beginning of the program, while it displayed an anabolic effect when added later in the presence of TGF-*β*1 [17]. Thus, the chondrogenic effects of FGF-18 on ADSCs remain to be further investigated. In addition, whether the combined application of FGF-18 and TGF-*β*3 would improve the efficiency of chondrogenic potential of ADSCs has not been thoroughly studied.

In the current study, we isolated human ADSCs and examined the main expression markers of adult MSCs. We evaluated the chondrogenic potential of FGF-18 on ADSCs by measuring glycosaminoglycan (GAG) content and ollagen amount, histologic appearance, and expression of cartilage-related genes. We also determined whether FGF-18 in combination with TGF-*β*3 could enhance chondrogenic differentiation of ADSCs in an *in vitro* pellet model.

## 2. Materials and Methods

### 2.1. Cell Isolation and Cultivation

All adipose tissues were obtained from three different donors with informed consent (age range 55–75 years). Ethical approval for this work was granted by the ethics committee of the No. 1 Affiliated Hospital of PLA General Hospital. The tissue was washed extensively with PBS, completely diced, and then digested with 0.1% collagenase A (Sigma, USA) solution at 37°C for 60 min. Then the digested tissue was filtered using a 75 *μ*m filter mesh (BD Biosciences, USA) and centrifuged at 1200 rpm for 5 min, and the supernatant was removed along with the mature adipocytes. Subsequently, cell pellets were resuspended in DMEM LG with 10% FBS (Corning, USA) and cultured on flasks in an incubator with 5% CO_2_, at 37°C. The medium was changed on the following day and thereafter every 3 days. When the cells reached 90% confluence, the cultures were trypsinized and passaged a further two times in T75 culture flasks (Corning, USA). Passage 3 to 5 cells were used for chondrogenic differentiation experiment.

### 2.2. Cell Characterization by Flow Cytometry

Human ADSCs from passage 3 were characterized by flow cytometry using the following monoclonal antibodies: anti-human CD14-PE, CD34-PE, CD45-PE, CD90-FITC, CD105-PerCP, and CD166-PE (BD Biosciences). Cells were digested with 0.25% trypsin/EDTA, resuspended with PBS/FBS(1%), counted, and adjusted to a density of 1 × 10^7^/mL. In each flow cytometric tube, aliquots containing 1 × 10^6^ ADSCs were incubated with antibodies for 1 h at 4°C away from light, according to the instructions. For negative control, the buffer was added with a mouse IgG1 antibody. The suspended ADSCs were washed and then analyzed with a FACSCalibur device (BD Biosciences). For each sample, 20,000 events were acquired and analyzed with the CellQuest software (BD Biosciences). Cells were considered MSCs if they were positive for CD90, CD105, and CD166 and negative for CD14, CD34, and CD45.

### 2.3. Chondrogenic Differentiation in Pellet Culture

High-density cell pellet cultures are used to examine the chondrogenic differentiation of MSCs. For the preparation of each pellet, passage 3~5 human ADSCs were harvested and counted and aliquots of 5 × 10^5^ cells in the defined medium were spun down at 300 g for 10 minutes in a 15 mL polypropylene conical tube (Corning, USA). The basic chondrogenic culture medium consists of DMEM supplemented with 10% FBS (Corning, USA), 100 nM dexamethasone, 1% ITS+ Premix, 100 *μ*g/mL sodium pyruvate, 50 *μ*g/mL L-ascorbate-2-phosphate, 40 *μ*g/mL proline, and 1% penicillin–streptomycin. All reagents were obtained from Sigma-Aldrich unless otherwise noted. Pellets were divided into 4 experimental groups, including control group, TGF-*β*3 group (10 ng/mL), FGF-18 group (100 ng/mL), and the combination of TGF-*β*3 and FGF-18 group. Pellets were cultured with the basic chondrogenic medium (see above) supplemented with TGF-*β*3 (PeproTech, USA) and/or FGF-18 (PeproTech, USA) for indicated periods. The control group was treated with the basic chondrogenic medium which is the same with that in the other three groups. The medium was changed every three days.

### 2.4. Glycosaminoglycan (GAG) and DNA Quantification

At the indicated time points, ADSC pellets were collected and digested for 18 h at 60°C using 125 *μ*g/mL papain solution (1 mL/sample papain solution) and then clarified by centrifugation. Aliquots of the supernatant were assayed separately for glycosaminoglycan (GAG) and DNA content. GAG in pellets was evaluated using the dimethylmethylene blue (DMMB) binding assay described before [18]. Two hundred microliters of DMMB reagent (16 mg of dye in 1 L of water containing 3.04 g glycine, 2.37 g NaCl, and 95 mL 0.1 M HCl) was added to 50 *μ*L of supernatant of papain digest, and the absorbance at 525 nm was immediately measured. The standard curve for the analysis was made using a chondroitin 4-sulfate standard. DNA was extracted from the papain-digested sample by the standard protocol using proteinase K and phenol, then the concentration was determined directly using NanoDrop ND 2000 (Thermo Scientific, USA). The DNA content per scaffold was calculated while taking into consideration sample dilution. For glycosaminoglycan synthetic activity, the ratio of GAG/DNA in each sample was normalized.

### 2.5. Hydroxyproline (HYP) Assay

Total collagen content was measured by first quantifying the hydroxyproline content in pellets after a 5-week chondrogenic induction. Hydroxyproline (HYP) assay was performed according to the instructions of the commercial kit (Nanjing Jiancheng Bioengineering Institute, China). Briefly, samples were hydrolyzed with an equal amount of 6 M HCl at 95°C for 5 h. Then, hydrolyzed samples were cooled to room temperature and the pH value was adjusted to 6.0–6.8, followed by an orderly addition of three reagents in the kit. After that, samples were incubated at 60°C for 15 min and centrifuged at 3500 rpm for 10 min. Supernatants were used to determine the absorbance at 550 nm. The hydroxyproline content was calculated according to the formula described in the instructions. The total amount of collagen per scaffold was converted by the content of hydroxyproline using a previously reported hydroxyproline to collagen ratio of 1 : 7.69 [19].

### 2.6. RNA Extraction, cDNA Synthesis, and Real-Time PCR

Total RNA was extracted from pellets using the TRIzol reagent according to the manufacturer's instructions (Invitrogen, USA). Before RNA isolation, human ADSC pellets were preserved in RNAlater RNA stabilization reagent after chondrogenic induction for 5 weeks (Qiagen, USA). Then, RNA was reverse-transcribed to cDNA using All-in-One cDNA Synthesis SuperMix (Bimake, USA). Real-time PCR was performed to measure the relative mRNA levels using QuantStudio 5 (Applied Biosystems, USA) with UltraSYBR Mixture (CWBIO, China). All samples were measured in triplicate and normalized to internal control GAPDH. The expression level of each target gene was calculated by QuantStudio Design and Analysis desktop software. The anneal temperature is 57°C, and primer sequences were described as follows: collagen II (forward-5′AAG TCC CTC AAC AAC CAG AT3′ and reverse-5′CCA GTA GTC TCC ACT CTT CCA3′), collagen X (forward-5′GGC AAC AGC ATT ATG ACC C3′ and reverse-5′GAT GAT GGC ACT CCC TGA A3′), Sox-9 (forward-5′GTA CCC GCA CTT GCA CAA C3′ and reverse-5′TTC TTC ACC GAC TTC CTC C3′), aggrecan (forward-5′TAC AAA CGC AGA CTA CAG AAG C3′ and reverse-5′AAA GCG ACA AGA AGA GGA CA3′), COMP (forward-5′CCC AGA AGA ACG ACG ACC AA3′ and reverse-5′CCC TAT ACC ATC GCC ATC ACT3′), Prg4 (forward-5′TGA CGG CTA TGA TTA CTA TGC3′ and reverse-5′AGT TGA CTC CTC CTT TGC TC3′), and GAPDH (forward-5′TGG CTA CAG CAA CAG GGT G3′ and reverse-5′ATG GCA ACT GTG AGG AGG G3′).

### 2.7. Histological and Immunohistochemical Analysis

After a 5-week chondrogenic induction, human ADSC pellets were fixed overnight in 4% paraformaldehyde (PFA), followed by dehydration in a graded ethanol series, and embedded in paraffin. The specimens were sliced into 5 *μ*m sections. The sections were stained with hematoxylin and eosin (H&E) or Alcian blue and Sirius red to visualize the presence and distribution of glycosaminoglycan (GAG) and total collagen as described [20]. Immunohistochemical examination was performed using a monoclonal antibody against type II collagen (Abcam, USA) as described previously [20], and cell nuclei were counterstained with hematoxylin. All images were obtained with a light microscope (Olympus, Japan) and a digital camera attachment (Nikon, Japan).

### 2.8. Statistical Analysis

Results are reported as means ± standard deviation. Statistical analysis was performed using SPSS 17.0 software. One-way ANOVA and Dunn's multiple comparison test were used to examine significant difference (*p* < 0.05).

## 3. Results

### 3.1. Immunophenotypic Characterization of Cultured ADSCs

**Figure 1:**
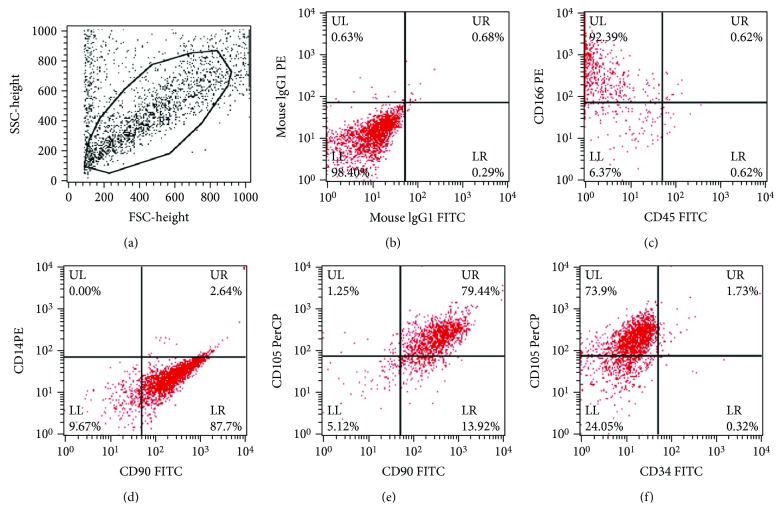
FACS analysis of ADSC characteristics. (a) The representative images of gating. (b) The negative results with the isotype-matched control antibodies. ADSC surface markers (CD90, CD105, CD166, CD14, CD 34, and CD45) were determined (c–f).

Human adipose-derived stem cells (ADSCs) were isolated from adipose tissue, and subsequently passages 3 to 5 were used in our study. Morphologically, cultures showed the typical fibroblast-like features as primary MSC (data not shown). Since mesenchymal stem cells were known to express typical surface markers, the phenotypes of ADSCs at passage 3 were characterized by flow cytometry analysis. In our study, mesenchymal characteristics were evaluated using the positive MSC markers, CD90, CD105, and CD166, and the negative MSCs markers, CD14, CD34, and CD45. As expected, the MSC markers CD90, CD105, and CD166 were observed in more than 75% of the cells, while the MSC negative markers CD14, CD34, and CD45 were observed in less than 5% of the cells (Figures 1(a)–1(f)). These results suggested that ADSCs we cultured maintained typical phenotypes of MSCs and could be used to investigate the chondrogenic differentiation.

### 3.2. Biochemical Analysis of Extracellular Matrix on ADSC Pellet

**Figure 2:**
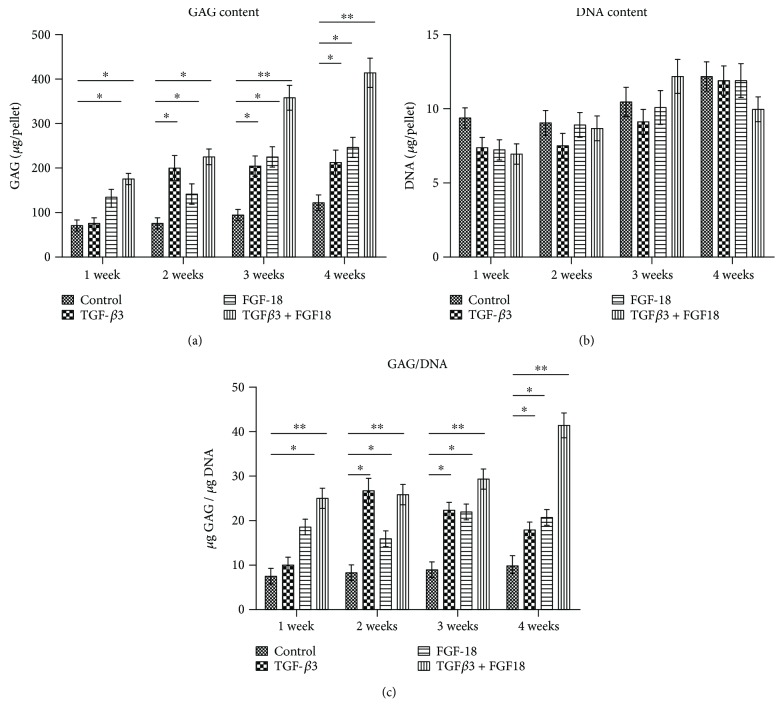
The quantification of GAG, DNA, and GAG/DNA in ADSC pellet induced by TGF-*β*3 alone, FGF-18 alone, and their combination at indicated time points. (a) Synthesis of sulfated GAG (sGAG) from hADSC pellet. (b) Change in hADSC DNA contents in pellet culture at different time points. (c) The change of GAG/DNA in ADSC pellet at different groups and indicated time points. Values are mean ± SEM, *n* = 5. The asterisk ∗ (*p* < 0.05) and asterisks ∗∗ (*p* < 0.01) indicate significant difference compared to the control group.

**Figure 3:**
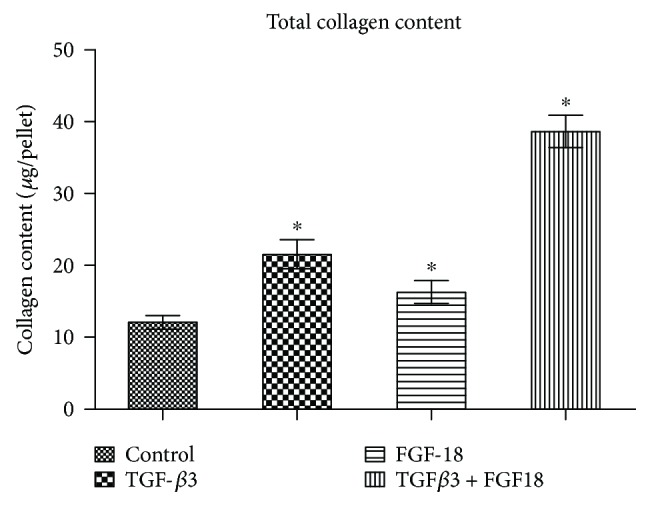
The quantification of collagen content per ADSC pellet after a 5-week chondrogenic induction. The asterisk ∗ (*p* < 0.05) indicates significant difference compared to the control group.

The 3D high-density pellet culture is a commonly used model for chondrogenesis in vitro. To investigate the chondrogenic potential of FGF-18 alone and in combination with TGF-*β*3, ADSCs were induced towards chondrocytes in the pellet culture using the basic chondrogenic medium supplemented with TGF-*β*3 only, FGF-18 only, or the combined application of FGF-18 and TGF-*β*3 for the indicated periods. ADSCs cultured in the basic chondrogenic medium were considered as negative control, while exposed TGF-*β*3 was considered as a positive group. For a quantitative comparison of matrix production between the different groups over time, glycosaminoglycan (GAG) levels and collagen content in ADSC pellets were examined by DMMB assay at 1, 2, 3, and 4 weeks and hydroxyproline quantification at 5 weeks, respectively. After a 2-week chondrogenic induction, TGF-*β*3 significantly increased the production of GAG as in previous reports (Figure 2(a)). Notably, pellets cultured with FGF-18 showed a remarkable increase in GAG content compared with the negative control at four different time points, suggesting that addition of FGF-18 throughout the culture period stimulated chondrogenesis of ADSCs as well as TGF-*β*3 (Figure 2(a)). In addition, highest GAG levels were observed in the combined treatment of TGF-*β*3 and FGF-18 group at the indicated time points (Figures 2(a) and 2(c)), suggesting that there were synergistic effects of FGF-18 and TGF-*β*3 on the chondrogenic differentiation of ADSCs. No significant difference in DNA content per pellet was observed among these groups (Figure 2(b)). GAG normalized to DNA was higher in the chondrogenic group containing the growth factor than in the negative control, regardless of the presence of TGF-*β*3 or FGF-18 (Figure 2(c)). There was nearly no increase in GAG/DNA in the control group over time (Figure 2(c)). Also, synergistic effects of FGF-18 and TGF-*β*3 on the production of GAG were observed in four different time points (Figure 2(c)). Quantification of collagen content in ADSC pellet induced for 5 weeks showed that TGF-*β*3 or FGF-18 alone enhanced collagen production compared with the control, respectively (Figure 3). In addition, TGF-*β*3 in combination with FGF-18 largely increased the average amounts of collagen per pellets and showed synergistic effects on ADSC chondrogenesis (Figure 3). A biochemical evaluation of the GAG and collagen content suggests that FGF-18 worked as a chondrogenic agent as well as TGF-*β*3, and a combination of FGF-18 and TGF-*β*3 would probably improve the chondrogenic differentiation potential of ADSCs.

### 3.3. Histological and Immunohistochemical Analysis of ADSC Pellet

**Figure 4:**
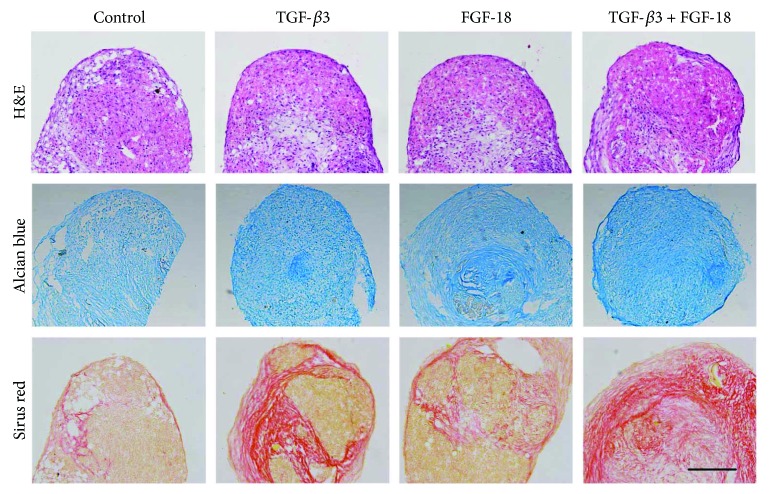
Histological appearance of hMSC pellets: (a) H&E staining; (b) Alcian blue staining; (c) Sirius red staining. Images are from representative cell cultures at 200 times magnification. Bar, 100 *μ*m.

**Figure 5:**
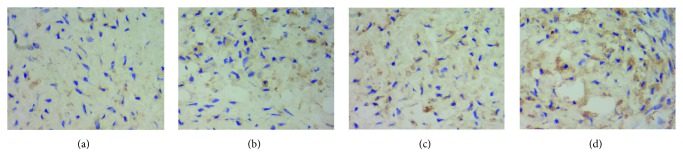
Immunohistochemical staining for type II collagen (ColII) expression in ADSC pellet induced to chondrogenic differentiation for 5 weeks: (a) control; (b) TGF-*β*3; (c) FGF-18; (d) FGF-18 + TGF-*β*3. Increased levels of type II collagen expression in pellet culture containing FGF-18 and TGF-*β*3 were detected by immunohistochemistry.

In order to evaluate the action of FGF-18 or combination of FGF-18 and TGF-*β*3 on ADSC chondrogenic differentiation, a histological and immunohistochemical evaluation was performed to determine the extracellular matrix synthesis and deposition in chondrogenic differentiated ADSCs in pellet culture. H&E staining showed no difference in pellet size and histological appearance in these pellets at 5 weeks (Figure 4(a)). Next, we performed Alcian blue and Sirius red staining for cartilage proteoglycan collagen, respectively. Alcian blue staining demonstrated that the blue coloration was increased in the extracellular matrix of ADSC cultures' exposure to FGF-18 and TGF-*β*3 compared to that of other cultures (Figure 4(b)). It also showed more GAG deposition in FGF-18-treated pellets compared with the negative control group (Figure 4(b)). After a 5-week chondrogenic induction, Sirius Red staining showed that a more intense staining signal for collagen was observed in the ADSC pellets treated with FGF-18 and TGF-*β*3 together (Figure 4(c)). We further determined the type II collagen expression level in ADSC pellets cultured for 5 weeks by immunohistochemical analysis. Consistent with the histological results, a higher type II collagen expression was found in the group of the combination of FGF-18 and TGF-*β*3 compared to that of other groups (Figures 5(a)–5(d)). There was also an increased expression of type II collagen in pellet cultures treated with FGF-18 or TGF-*β*3 alone compared with the negative control (Figures 5(a)–5(c)). These results further confirmed that FGF-18 has chondrogenic property as well as TGF-*β*3 and that FGF-18 in combination with TGF-*β*3 greatly potentiated a chondrogenic differentiation of human ADSCs in the *in vitro* pellet model.

### 3.4. Gene Expression Changes on ADSC Pellet

**Figure 6:**
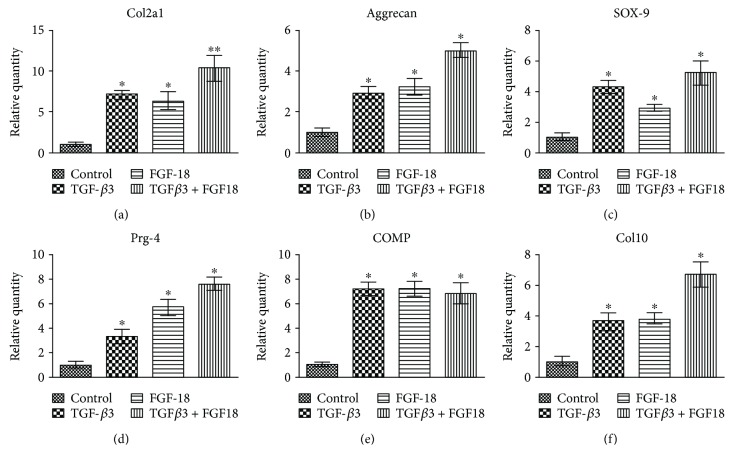
Gene expression analysis of the chondrogenic genes in ADSC pellets. Chondrocytic-related genes of Col2a1 (a), Aggrecan (b), SOX-9 (c), Prg4 (d), COMP (e), and Col10 (f) were quantified by real-time PCR in ADSC pellet, which were stimulated with TGF-*β*3 alone, FGF-18 alone, and their combination for 5 weeks. The asterisk ∗ (*p* < 0.05) and asterisks ∗∗ (*p* < 0.01) indicate significant difference compared to the control group.

To uncover the underlying gene expression pattern, the extent of chondrogenesis was evaluated by quantifying the chondrocytic cell-specific genes, including type II collagen (Col2a1), aggrecan, SOX-9, proteoglycan 4 (Prg4), cartilage oligomeric matrix protein (COMP), and type X collagen (Col10). After 5 weeks of chondrogenic induction, real-time PCR analysis found that all of these chondrocyte-related genes were much more highly expressed in ADSC pellets cultured in the presence of growth factors (FGF-18 alone, TGF-*β*3 alone, or FGF-18 + TGF-*β*3) than that in the negative control (Figures 6(a)–6(f)). Treatment with FGF-18 alone substantially upregulated chondrogenic gene expression relative to the negative control, similar to the effects of the well-known chondrogenic agent TGF-*β*3 (Figures 6(a)–6(f)). No significant difference in COMP expression was found between FGF-18 + TGF-*β*3 and FGF-18 or TGF-*β*3 alone (Figure 6(e)). However, the results revealed that the Col2a1, aggrecan, SOX-9, Prg4, and Col10 mRNA expression in the FGF-18- and TGF-*β*3-cotreated pellets was significantly higher than that in pellets treated with FGF-18 or TGF-*β*3 alone, suggesting that there were synergistic effects of FGF-18 and TGF-*β*3 on chondrogenic gene expression in the *in vitro* pellet model (Figures 6(a)–6(d) and 6(f)).

## 4. Discussion

In this study, we had successfully isolated human ADSCs and characterized the expression of their surface antigens. We found that FGF-18, similarly to TGF-*β*3, has a positive impact on chondrogenic differentiation as well as matrix deposition when present throughout the culture time. In addition, we observed synergistic effects of FGF-18 and TGF-*β*3 on the chondrogenic differentiation of ADSCs.

Human ADSCs were considered as an optional alternative for cell-based articular cartilage repair in the clinic because they were abundant in supply and possessed chondrogenic potential and easy accessibility [21]. ADSCs show similar characteristics with BMSCs, but they also exhibit a number of different features, that is, cell surface markers, differentiation potential, and abundance in the body [4]. In the current study, we isolated ADSCs from human adipose tissue and analyzed the cell surface markers for undifferentiated ADSCs. Consistent with the results described previously [22], we observed that our cultured ADSC populations were positive for CD90, CD105, and CD166 and negative for CD14, CD34, and CD45, suggesting that these ADSCs fitted with the criteria of MSCs and could be further used for chondrogenic differentiation analysis using an *in vitro* pellet culture model.

A major challenge in ADSC application for cartilage repair is on how to control and facilitate their chondrogenic differentiation. Despite growing information regarding MSCs, the mechanisms contributing to the chondrogenesis of ADSCs are not well defined. One rational strategy is to identify key growth factors and recapitulate the in vivo environment by using these growth factors to improve chondrogenesis of ADSCs. Previous studies have suggested that various growth factors such as TGF-*β*s, IGF-1, insulin, and BMPs were capable of stimulating in vitro chondrogenesis of ADSCs [8, 23–25]. In our pellet chondrogenic culture system, as a positive group, TGF-*β*3-treated ADSC pellets also showed an increased total amount of GAG at different times, a rise in collagen expression, and an upregulated expression of chondrocyte-related genes after a 5-week chondrogenic induction. Importantly, our results revealed that FGF-18 greatly upregulated the production of the extracellular matrix of GAG and collagen by biochemical evaluation and histological analysis, suggesting that FGF-18 alone supplemented with the basic chondrogenic medium is sufficient to direct chondrogenic commitment of ADSCs as well as TGF-*β*3 when present throughout the entire differentiation program. It was consistent with the results that FGF-18 promoted chondrogenic differentiation and cartilage production in limb bud mesenchymal cells [13].

There are two different opinions about the role of FGF-18 in MSC chondrogenic differentiation: one thinks that FGF-18 positively regulates chondrogenic differentiation [12, 13], and another suggests that FGF-18 negatively regulates chondrogenesis [26]. Our results showed that FGF-18 promotes chondrogenic differentiation of ADSCs by increasing the expression of SOX-9 and also promotes the expression of chondrocytic matrix gene Col2a1 and aggrecan during chondrogenesis of ADSCs. Besides that, the timing point of FGF-18 action is also controversial. Some reports suggested that FGF-9 and FGF-18 inhibited extracellular production or had no significant effects at the early stage of chondrogenesis, but played an anabolic effect when added at the late stage [17, 27]. In contrast with these reports, we found that FGF-18 had potent chondroinductive actions on MSCs when present in the chondrogenic medium at the entire culture period. The contradictory results might be related to different chondrogenic induction media (with or without 10% FBS), the concentration of FGF-18 (100 ng/mL or 10 ng/mL), inherent differences of ADSCs, and so on. The chondrogenic culture medium used in our study contained 10% FBS, and that might be enough to stimulate the proliferation and survival of ADSCs in the pellet culture model and cover or exceed the effects of growth factors on ADSC proliferation. In addition, we inferred that higher concentration of FGF-18 (100 ng/mL) may also have stronger chondrogenic potency for ADSCs than the previously used 10 ng/mL FGF-18 [17].

To our knowledge, this study firstly showed the enhanced and synergistic effects of FGF-18 in combination with TGF-*β*3 on chondrogenesis of ADSCs. When ADSC pellets were subjected to a combination of FGF-18 and TGF-*β*3 throughout the culture period, more chondrocytic markers and extracellular matrix were produced. ADSC pellets cotreated with these two growth factors displayed increased GAG accumulation, improved histologic appearance with intense Alcian blue and Sirius red staining signal, and increased transcription of cartilage-related genes, such as aggrecan, Sox-9, Col2a1, and Prg4. Previously, some results also showed that insulin-like growth factor 1 (IGF-1) enhanced the chondrogenesis effects of TGF-*β*1 and the combined application of TGF-*β*3 and BMP-2 has a synergistic effect on chondrogenic differentiation [23, 25]. In addition, it also showed that BMP-2 and IGF-1 were capable of modulating the proliferation and chondrogenic differentiation of ADSCs [28]. In the current study, we found that FGF-18 and TGF-*β*3 had combined effects on inducing differentiation of ADSCs into chondrocytes with a histological appearance and chondrogenic gene expression profiles in the *in vitro* pellet model. The specific molecular mechanisms of synergism need to be further elucidated in the near future study. In addition, whether these two growth factors can promote chondrogenesis in vivo remains to be further investigated.

## 5. Conclusions

In conclusion, our results from ADSC pellet culture demonstrated that chondroinductive actions of FGF-18 were nearly equal to those of TGF-*β*3, and the two growth factors had synergistic effects on ADSC chondrogenesis. Therefore, simultaneous stimulation of ADSCs with TGF-*β*3 and FGF-18 may serve as a useful approach for assisting translational research for cell-based therapies targeting cartilage tissue regeneration.
